# Screening of single nucleotide polymorphisms within HLA region related to hematopoietic stem cell transplantation using MassARRAY technology

**DOI:** 10.1038/s41598-023-33149-4

**Published:** 2023-04-11

**Authors:** Shu-Hui Tsai, Pi-Yueh Chang, Ying-Hao Wen, Wei-Tzu Lin, Fang-Ping Hsu, Ding-Ping Chen

**Affiliations:** 1 Department of Laboratory Medicine, Linkou Chang Gung Memorial Hospital, Taoyuan, 333 Taiwan; 2 Department of Medical Biotechnology and Laboratory Science, College of Medicine, Chang Gung University, Taoyuan, Taiwan; 3 Graduate Institute of Clinical Medical Sciences, College of Medicine, Chang Gung University, Taoyuan, Taiwan

**Keywords:** Biotechnology, Molecular biology, Genetic markers

## Abstract

A growing number of studies showed that single nucleotide polymorphisms (SNPs) in the human leukocyte antigen (HLA)-related genes were associated with the outcome of hematopoietic stem cell transplantation (HSCT). Thus, other SNPs located nearby the classical HLA genes must be considered in HSCT. We evaluated the clinical feasibility of MassARRAY by comparing to Sanger sequencing. The PCR amplicons with each one of the 17 loci that were related to the outcomes of HSCT published by our previous study were transferred onto a SpectroCHIP Array for genotyping by mass spectrometry. The sensitivity of MassARRAY was 97.9% (614/627) and the specificity was 100% (1281/1281), where the positive predictive value (PPV) was 100% (614/614) and the negative predictive value (NPV) was 99.0% (1281/1294). MassARRAY is high-throughput, which can accurately analyze multiple SNPs at the same time. Based on these properties, we proposed that it could be an efficient method to match the genotype between the graft and the recipient before transplantation.

## Introduction

In the past, before receiving hematopoietic stem cell transplantation (HSCT), the human leukocyte antigen (HLA) alleles between the recipient and the donor must be verified that they were matched in order to prevent transplant rejection. Although the transplantation of unrelated cord blood and unrelated related donors has become an effective alternative, due to the limited source of matched related or unrelated doners, haploidentical HSCT still suffers from the risk of GVHD and other poor outcomes^1^. And the large studies which have shown that transplant outcomes using haploidentical donors do not have elevated rates of GVHD and poor survival^2^.

In clinical, transplant failure may still exist even if the hematopoietic stem cell with the same HLA type is chosen for transplantation, and graft versus host disease (GVHD) is also frequently observed^3,4^. It was indicated that the effectiveness of HSCT may regulated by other factors in addition to the HLA system. A review literature showed that minor histocompatibility antigens and non-HLA single-nucleotide polymorphisms (SNPs) involved in immune regulation, as well as microRNAs regulating these SNPs, all affect transplantation outcomes^5^.

We observed that there were 74.5% of acute GVHD and 29.1% of relapse in HLA-matched HSCT cases that were typed through next generation sequencing (NGS)^6^. According to this funding, Chen, et al. surmised that may not be due to the insufficient resolution of HLA typing. In order to clarify it, Chen, et al. analyzed the correlation between single nucleotide polymorphisms (SNPs) located near the classical HLA genes, encoded by chromosome 6, and the adverse outcomes of bone marrow transplantation (BMT) and cord blood transplantation (CBT). It was found that several SNPs within the HLA region were statistically significant with the outcomes of HSCT. In the BMT cases, it was found that the 11 SNPs were associated with relapse, survival, or GVHD, including the rs2518028 of *HCP5*, the rs213210 and rs107822 of *RING1*, the rs17220087 and the rs2070120 of *HLA-DOB*, the rs1536215 and the rs139791445 of *TRIM27*, the rs79327191 of *HLA-DOA*, the rs111394117 of *NOTCH4*, the rs3130048 of *BAG6*, and the rs2009658 of *LTA*^6,7^. In the CBT cases, it was found that 11 SNPs were related to the outcomes, including the rs2518028 and the rs4713466 of *HCP5*, the rs17220087 and rs2070120 of *HLA-DOB*, the rs5009448, rs435766, and rs2523958 of *MICD*, the rs213210 and rs107822 of *RING1*, the rs986522 of *COL11A2*, and the rs9276982 of *HLA-DOA*^8,9^, where the rs2518028, the rs213210, the rs107822, the rs17220087, and the rs2070120 had significance in both BMT and CBT analysis. These SNPs either present in the donor DNA, in the recipient DNA, or were mismatched between the donor and the recipient DNA leading to favorable or unfavorable post-HSCT clinical outcome of the recipients. For details, please refer to reference number 3 to 6. It was conjectured that these SNPs may affect the immune response, leading to the worse outcomes of post-HSCT. Thus, when matching the genotype between the graft and the recipient, we cannot only focus on the classical HLA genes, the SNPs within the HLA region must be considered together.

Nowadays, Sanger sequencing is the gold standard for SNP analysis in clinical molecular laboratories, but it has limited ability to analyze multiple gene loci at a time. Thus, it should be completed by several PCR attempts in Sanger sequencing, which is complex and cumbersome. Additionally, 1 μL of DNA samples will be consumed for each PCR reaction, which is not suitable for cases where the sample is difficult to obtain. Because Sanger sequencing is cumbersome, time-consuming, and needs a lot of samples for analyzing multiple loci, it was not suitable for analyzing the HSCT outcomes-related SNPs. In recent years, mass spectrometry technology has rapidly developed, making the MassARRAY method gradually become a key technology for biological analysis.

MassARRAY has the advantages of high throughput, high cost-performance, high sensitivity, and high flexibility^10^. In the MassARRAY method, a chip has 96 wells, which can detect up to 96 samples at the same time. In addition, up to 40 SNPs can be detected in one well, and it only needs 10 ng of DNA to test, which can shorten the time and make good use of the precious samples^11^. The principle of MassARRAY is to directly detect the mass-to-charge ratio of the extended products, in which the designed primer does not require any fluorescence or protein calibration, so it can lower the cost. Furthermore, MassARRAY has high flexibility of scale with versatility, and the numbers and loci of samples on a chip can be selected at will.

MassARRAY has been applied in clinical for many tests, such as antimicrobial resistance detection, Cytochrome p450 genotype identification, methylation analysis, and so on^12,13^. Moreover, it can also be used for SNP identification^14^. MassARRAY system is to analyze the ions converted from nucleic acid by matrix-assisted laser desorption/ionization-time of flight mass spectrometry (MALDI-TOF MS) technology. The sample molecules are ionized and enter the time-of-flight tube containing an electric field, then the ions are separated according to the mass-to-charge ratio. The smaller the ion mass is, the shorter the flight time is. Finally, after all the ions arrived at the detector, a signal will be generated to identify the results of genotype analysis^15^. In order to rapidly analyze the SNPs associated with the outcomes of post-HSCT. In this study, a total of 17 SNP sites with significant differences in *p* values published in the past were selected and combined with the MassArray platform to design a detection set related to the effect after transplantation^6–9^. SNP analysis was carried out on 30 pairs of donors and recipients through MassArray platform, and the results were compared with Sanger's sequencing. We developed a panel for diagnosis these 17 SNPs that have already been described, using the MassARRAY iPLEX system. The aim of this study was to evaluate the accuracy of the MassARRAY method by comparing it with Sanger sequencing based on the 17 SNPs published in the previous. In addition, the clinical feasibility of MassARRAY were also evaluated.

## Results

**Figure 1:**
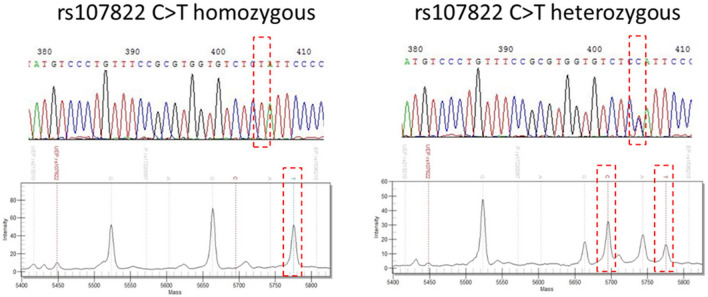
The comparisons of MassARRAY and Sanger sequencing. The y-axis of MassARRAY plot is referred to peak intensity and the x-axis of MassARRAY plot is referred to mass (daltons). Mass spectrum of a sample showing a single peak of 5785 Da (left) indicating TT genotype. Mass spectrum of a sample showing peaks at both 5695 Da and 5785 Da indicating CT genotype.

A total of 60 samples, 30 pairs of recipients and their corresponding donors, were tested by both Sanger sequencing and MassARRAY. The 17 significant SNPs were tested through these two methods. The comparison of the results between MassARRAY and Sanger sequencing were shown in Fig. 1, in which Sanger sequencing was used as a reference result to compare with MassARRAY (Table 1). It was found that the accuracy of the MassARRAY method was 100% in the 13 SNPs: rs139791445 and rs1536215 of *TRIM27*; rs2070120 of *HLA-DOB*; rs79327197 and rs9276982 of *HLA-DOA*; rs3130048 of *BAG-6*; rs2009658 of *LTA*; rs2518028 and rs4713466 of *HCP5*; rs2523958 and rs435766 of *MICD*; rs986522 of *COL11A2*; rs111394117 of *NOTCH4*. And the accuracy was 95–98% in 3 SNPs: rs107822 and rs213210 of *RING1*; rs17220087 of *HLA-DOB*. The slightly lower accuracy (89%) was only seen in one SNP, rs5009448 of *MICD*.

**Table 1:** Comparing the analysis data of MassARRAY and Sanger sequencing.

Gene	SNP		MassARRAY data and Sanger sequencing								Accuracy
			Matched				Unmatched				
			BMT		CBT		BMT		CBT		
			PT	DN	PT	DN	PT	DN	PT	DN	
RING1	rs107822		N = 24	N = 24	N = 5	N = 5	N = 0	N = 0	N = 1	N = 1	
		CC	3	2	0	0	0	0	1	1	96%
		CT	11	10	2	1					
		TT	9	11	2	3					
		n/a	1	1	1	1					
RING1	rs213210		N = 23	N = 24	N = 6	N = 5	N = 1	N = 0	N = 0	N = 1	
		AA	3	3	0	0	1	0	0	1	96%
		AG	11	12	4	1					
		GG	8	8	1	2					
		n/a	1	1	1	2					
TRIM27	rs139791445		N = 24	N = 24	N = 6	N = 6	N = 0	N = 0	N = 0	N = 0	
		CC	22	23	5	4	0	0	0	0	100%
		CG	1	0	0	1					
		GG	0	0	0	0					
		n/a	1	1	1	1					
TRIM27	rs1536215		N = 24	N = 24	N = 6	N = 6	N = 0	N = 0	N = 0	N = 0	
		CC	20	16	4	2	0	0	0	0	100%
		CG	3	6	1	2					
		GG	0	1	0	1					
		n/a	1	1	1	1					
HLA-DOB	rs17220087		N = 24	N = 21	N = 6	N = 6	N = 0	N = 3	N = 0	N = 0	
		AA	0	0	0	0	0	3	0	0	95%
		AC	1	0	0	0					
		CC	22	20	5	5					
		n/a	1	1	1	1					
HLA-DOB	rs2070120		N = 24	N = 24	N = 6	N = 6	N = 0	N = 0	N = 0	N = 0	
		AA	0	0	0	0	0	0	0	0	100%
		AG	1	3	0	0					
		GG	23	20	5	5					
		n/a	0	1	1	1					
HLA-DOA	rs79327197		N = 24	N = 24	N = 6	N = 6	N = 0	N = 0	N = 0	N = 0	
		AA	23	22	5	4	0	0	0	0	100%
		AG	0	1	0	1					
		GG	0	0	0	0					
		n/a	1	1	1	1					
HLA-DOA	rs9276982		N = 24	N = 24	N = 6	N = 6	N = 0	N = 0	N = 0	N = 0	
		AA	2	1	0	1	0	0	0	0	100%
		AG	6	5	2	1					
		GG	15	17	3	3					
		n/a	1	1	1	1					
BAG-6	rs3130048		N = 24	N = 24	N = 6	N = 6	N = 0	N = 0	N = 0	N = 0	
		CC	1	3	1	1	0	0	0	0	100%
		CT	11	11	3	0					
		TT	11	9	1	4					
		n/a	1	1	1	1					
LTA	rs2009658		N = 24	N = 24	N = 6	N = 6	N = 0	N = 0	N = 0	N = 0	
		CC	17	17	2	4	0	0	0	0	100%
		CG	7	6	3	1					
		GG	0	0	0	0					
		n/a	0	1	1	1					
HCP5	rs2518028		N = 24	N = 24	N = 6	N = 6	N = 0	N = 0	N = 0	N = 0	
		CC	20	18	3	3	0	0	0	0	100%
		CT	3	3	2	2					
		TT	1	2	0	0					
		n/a	0	1	1	1					
HCP5	rs4713466		N = 23	N = 24	N = 6	N = 6	N = 1	N = 0	N = 0	N = 0	
		CC	9	12	3	3	1	0	0	0	98%
		CT	11	10	2	2					
		TT	2	1	0	0					
		n/a	1	1	1	1					
MICD	rs2523958		N = 24	N = 24	N = 6	N = 6	N = 0	N = 0	N = 0	N = 0	
		AA	1	1	0	0	0	0	0	0	100%
		AG	8	8	0	1					
		GG	14	14	5	4					
		n/a	1	1	1	1					
MICD	rs435766		N = 24	N = 24	N = 6	N = 6	N = 0	N = 0	N = 0	N = 0	
		AA	2	3	0	0	0	0	0	0	100%
		AG	12	10	4	4					
		GG	9	10	1	1					
		n/a	1	1	1	1					
MICD	rs5009448		N = 23	N = 23	N = 3	N = 5	N = 1	N = 1	N = 3	N = 1	
		CC	10	12	2	3	1	1	3	1	89%
		CT	0	0	0	1					
		TT	13	10	0	0					
		n/a	0	1	1	1					
COL11A2	rs986522		N = 24	N = 24	N = 6	N = 6	N = 0	N = 0	N = 0	N = 0	
		CC	0	1	0	0	0	0	0	0	100%
		CG	9	9	1	1					
		GG	14	13	4	4					
		n/a	1	1	1	1					
NOTCH4	rs111394117		N = 24	N = 24	N = 6	N = 6	N = 0	N = 0	N = 0	N = 0	
		AA	0	0	0	0	0	0	0	0	100%
		AG	0	1	0	0					
		GG	23	22	4	5					
		n/a	1	1	2	1					

BMT: bone marrow transplantation; CBT: cord blood transplantation; n/a: not applicable; PT: patient; DN: donor.

Based on these 60 samples, we analyzed a total of 1,908 SNPs. The Sanger sequencing was used as gold standard and compared with the results of MassARRAY. We found that the sensitivity of MassARRAY was 97.9% and the specificity was 100%, where the positive predictive value (PPV) was 100% and the negative predictive value (NPV) was 99.0%. The detail results were shown in Table 2. Additionally, we compared the clinical benefit of these two methods. The comparison table (Table 3) demonstrated that MassARRAY can complete the results of these 17 loci in one test, and only needs 500 NT dollars (about USD $ 16.4). However, there are 10 tests of Sanger sequencing must be done to analyze these 17 SNPs, which takes a long time, and the cost is up to 2000 NT dollars (about USD $ 65.5). In addition, the other SNP detection method, real-time PCR, was compared together. Although the cost of MassARRAY and real-time PCR were similar, the times of real-time PCR were far from MassARRAY. Thus, MassARRAY seem to be the most efficient method for detecting so many SNPs.

**Table 2:** Comparing the result of MassARRAY and Sanger sequencing in 1,908 tests.

Result of new method MassARRAY	Result of Sanger sequencing		Total
	Variant allele	Reference allele	
Variant allele	614	0	PPV = 614/(614 + 0) = 100%
Reference allele	13	1281	NPV = 1281/(13 + 1281) = 99.0%
	Sensitivity = 614/(614 + 13) = 97.9%	Specificity = 1281/(1281 + 0) = 100%	

PPV: positive predictive values; NPV: negative predictive values.

**Table 3:** Comparison of different methods for detecting these 17 SNPs.

	MassARRAY iPLEX	Sanger sequencing (standard PCR)	Real-time PCR
PCR / 17 SNPs	Once	10 times	17 times
Cost / 1 test	NT $ 500 (about USD $ 16.4)	NT $ 200 (about USD $ 6.5)	NT $ 30 (about USD $ 1)
Total cost	NT $ 500 (about USD $ 16.4)	NT $ 2000 (about USD $ 65.5)	NT $ 510 (about USD $ 16.7)

Sanger sequencing requires individual experiments and analysis for each gene locus. There were 10 sequencing reactions required to analyze these 17 mutation loci. It cost 200 NT dollars (about USD $ 6.5) each time for standard PCR. Thus, the cost of Sanger sequencing is 2000 NT dollars, and the experimental process is time-consuming and labor-consuming. However, MassARRAY can simultaneously analyze 17 variation sites in one reaction, with a detection cost of 500 NT dollars. The sample size required for analysis is small, and the detection results can be obtained in one day. MassARRAY has both high throughput and high-cost performance. For the test items that need to detect multiple SNPs at the same time. Thus, it is more in line with clinical benefits than the traditional sequencing method.

## Discussion

MassARRAY has been used in various SNP genotyping studies. In 2015, Mollinari et al. published that accurate genotyping played an important role in the construction of genetic maps and the implementation of genome assembly of polyploid species. They found that MassARRAY could accurately estimate the number of individuals of a specific species in the ecosystem with different alleles in a certain region even when the polyploidy was unknown^16^. Additionally, in 2019, Liu et al. compared the results of MassARRAY and pyrosequencing for clopidogrel efficiency genotyping, and it was shown that MassARRAY could be an outstanding tool for genotyping the SNPs in patients that were potential candidates for targeted therapies^17^. Moreover, Nyasinga et al. believed that iPlex MassARRAY could be used as a useful monitoring tool for genotyping of *Staphylococcus aureus* isolates in Africa, so it was a faster, more affordable, and quite accurate method to identify genotypes with clinical significance^18^. These studies all showed that MassARRAY was a feasible method for genotyping, and it could even replace other sequencing methods in clinical. As for our results, we found that the analysis results of MassARRAY and Sanger sequence were highly consistent. Comparing to the data of Sanger sequencing, the sensitivity of MassARRAY was 100%, the specificity was 98.9%, the PPV was 99.6%, and the NPV was 98.9%. The 100% sensitivity is indicated that the MassARRAY can be a reliable platform for detection. The specificity refers to the ability of the test to correctly identify which loci without mutation (true negative rate). The specificity was 98.1%, in other words, it has 1.9% chance of making a wrong judgment. It was indicated that there are 1–2 times of misjudgments per 100 analyses. The PPV and NPV refer to the proportions of positive/ negative results in the tests that are truly positive/ negative. The PPV was 99.6%, which is indicated that the proportion of misjudgment is less than once in every 100 test results. The NPV was 98.9%, in other words, it has 1.1% chance of making a wrong judgment.

For detecting the panel of these 17 SNP, it can be done in one reaction of MassARRAY method, and the cost was about 500 NT dollars. By contrast, Sanger sequencing was relatively time-consuming and high-cost. Additionally, the advantage of MassARRAY is not only its lower cost, but also its ability to analyze multiple sites at one time and its high flexibility that allowing you to choose the loci you want to analyze at will. If there are new effective loci in the future, it can be immediately added in the panel to make the analysis more comprehensive and perfect. At present, most research institutions related to genetic medicine have also used MassARRAY system to test the large samples.

In this study, we suggested that MassARRAY is a fast, economical, and accurate platform for SNP detection, which could be used as an excellent tool for selecting the best donor for HSCT. We expected that it could accelerate the time of matching in clinical and improve the survival rate of transplant cases. People are interested in rapid, reliable, and accurate mutation screening methods. The advantage of MassARRAY is that it can detect multiple SNPs in a test and determination of the same sample, which means that fewer samples and shorter time are required to screen different gene loci in DNA.

In summary, we proposed the 3 advantages of MassARRAY for detecting multiple mutations: (1) The specificity of MassARRAY was 98.9%. This may be related to the high sensitivity of mass spectrometry, which can remedy the low sensitivity of Sangar sequencing. (2) It can reduce the detection cost and provide a direct economic benefit in clinical application. (3) It has high throughput and can detect the SNP loci of HSCT related adverse reactions at one time, which can optimize clinical resources. Therefore, MassARRAY is an efficient technology that provides a highly sensitive method for molecular classification.

## Limitation

According to the results, a large part of the data was consistent between these two methods, and only a few MassARRAY data was different from Sanger sequencing. This may be because the sample has been stored for a long time, resulting in a decrease in concentration, which was prone to slight errors. In addition, it could have some technical error. For example, the specific of the primer is not specific enough, resulting in a sequencing error. That could be improved in the future. The disease-related SNPs were various between ethnicity, so the data used in here was only suited for Taiwanese population. In addition, Chen, et al. will continue to include cases to increase the credibility of the previous SNP results^6–9^.

## Materials and methods

### Study subjects

The study has been reviewed and approved by the Institutional Review Board (IRB) of Chang Gung Memorial Hospital (CGMH), and the approved number was 202000409B0. All the participants provided written informed consents to participate in this study, and all the methods were performed in accordance with relevant guidelines and regulations. In this study, a total of 30 HSCT donor-recipient pairs (30 pairs of recipient and donor samples, 60 pieces in total) were recruited in Chang Gung Memorial Hospital, in which 24 cases received peripheral blood stem cell transplantation (PBSCT) and BMT (acute myeloid leukemia: 11 cases; acute lymphoblastic leukemia: 13 cases) and 6 received CBT. The characteristics of these participants were shown in Table 4. The subjects used to study the SNPs involved donor- recipient pairs that were 12/12 HLA-matched.

**Table 4:** The characteristics of the cases receiving HSCT.

Case	PBSCT and BMT	24
	CBT	6
Sex	Female	15
	Male	15
Survival	Survival	24
	MSD-BMT	6
	MSD-PBSCT	2
	MUD-BMT	4
	MUD-PBSCT	5
	MUD-CBT	6
	Haplo-BMT	1
	Dead	6
	MSD-PBSCT	3
	MUD-BMT	1
	MUD-PBSCT	1
	Haplo-BMT	1
GVHD	Grade I-II	16
	MSD-BMT	3
	MSD-PBSCT	4
	MUD-BMT	4
	MUD-PBSCT	3
	MUD-CBT	2
	Grade III-IV	7
	MSD-BMT	2
	MSD-PBSCT	1
	MUD-PBSCT	1
	MUD-CBT	2
	Haplo-BMT	1
	Chronic	2
	MSD-BMT	1
	Haplo-BMT	1
	Non-GVHD	5
	MUD-BMT	1
	MUD-PBSCT	2
	MUD-CBT	2
CMV	CMV-infection	12
	Non-infection	18

### Sample preparation

Genomic DNA was extracted from peripheral blood by using QIAamp DNA Blood Mini Kit (Qiagen, Germany). The purity and concentration of the samples were checked using a NanoDrop^®^ 2000 spectrophotometer (Thermo Scientific, USA).

### Sanger sequencing technology

In the published literatures^6–9^, the 10 pairs of primers were designed to amplify the fragment ranged from 500 bp upstream to 500 bp downstream of selected SNPs and found out the 17 HSCT-related SNPs (rs107822, rs111394117, rs139791445, rs1536215, rs17220087, rs2009658, rs2070120, rs213210, rs2518028, rs2523958, rs3130048, rs435766, rs4713466, rs5009448, rs79327197, rs9276982, and rs986522), those SNPs have been uploaded to NCBI dbSNP repository (Supplementary Information). The primer sequences for amplifying the segment covering these 17 SNPs were shown in ref.^6–9^. The 25 μl of PCR mixture each contained 10 μM of forward and reverse primer, 8 μl of HotStart Taq DNA polymerase (Agilent, Santa Clara, California, USA), 50 ng of DNA sample, and 14 μl deionized water. After PCR amplification, the 5 μl of the PCR products were loaded in 1.5–2% agarose gel to confirm the size of the PCR fragment. After that, the remaining PCR products were purified by the Exonuclease I (Exo I) and recombinant Shrimp Alkaline Phosphatase (rSAP), then it was subject to direct sequencing.

### MassARRAY technology

**Figure 2:**
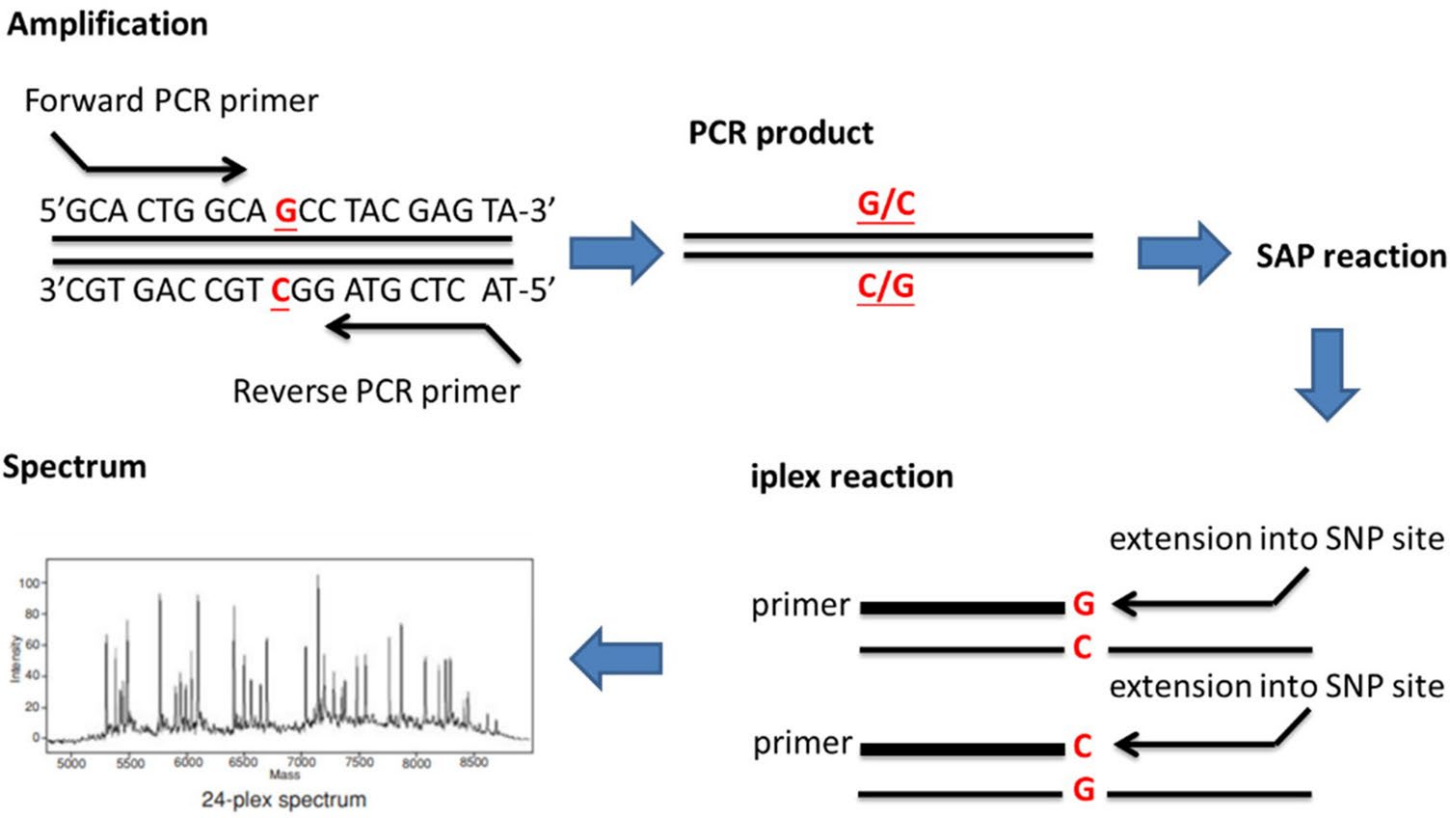
Experiment steps in genotyping using MassARRAY system. (a) Locus-specific amplification reaction. (b) Use SAP enzyme to unincorporated dNTPs. (c) Locus-specific primer extension reaction. The single base extension using mass-modified dideoxy-nucleotide terminators of an oligonucleotide primer. (d) Mass spectrum. By measuring the time-of-flight, the software will automatically analyze the mass of the analytes into a genotype.

The schematic diagram of MassARRAY method was shown in Fig. 2. The primers of the above-mentioned 17 SNPs used for MassARRAY were designed with Assay Design Suite software (Agena Bioscinece) (Table 5). The genotypes are detected using matrix-assisted laser desorption ionization mass spectrometry (MALDI-TOF). Following locus-specific PCR reaction, the PCR products were treated by ExoI enzyme to remove the remaining dNTP and primer, then proceed to the single base extension using mass-modified dideoxy-nucleotide terminators of an oligonucleotide primer (Table 6), which anneals upstream of the polymorphic site of interest. In the process of analysis, the crystals are formed by samples in SpectroCHIP, then the crystals are excited by a high-energy laser leading to DNA molecules ionization. Next, the ionized DNA samples enter a time-of-flight tube with an electric field. By measuring the time-of-flight of analytes by the detector, we can understand the genotype of the analytes.

**Table 5:** The primers of the 17 SNPs used for the amplification step of MassARRAY.

SNP_ID	PR	PF
rs2518028	ACGTTGGATGGCTGCATTATAAGGGTGAGG	ACGTTGGATGAGAGAAGGCTCGCCTTTTCC
rs435766	ACGTTGGATGTCTGTCCCCACTGGATCTG	ACGTTGGATGTTTCTGTGTGGGCTGAGTGC
rs213210	ACGTTGGATGAAAGATCTGCCGCTTTAGCC	ACGTTGGATGCAGGGTGGTAAGGGGAATC
rs107822	ACGTTGGATGTGTGTATGTCCCTGTTTCCG	ACGTTGGATGTTTGGACAATCAGGAGCCGC
rs17220087	ACGTTGGATGGGAGTAAGGTTGCTGCTGTC	ACGTTGGATGCACAGTGATGAAGGTCTCAG
rs1536215	ACGTTGGATGCCAACCTTGAAATGAGTCCC	ACGTTGGATGCAATCTCTGTTACTCTCACG
rs2070120	ACGTTGGATGGGGAAGAGAGTTATTCCCAG	ACGTTGGATGTAGTGATGAGTAGTCTGGGC
rs79327197	ACGTTGGATGTGGTTCTCGGGTAGTCTGTG	ACGTTGGATGGTCTGCCCCACTTAAAATAG
rs111394117	ACGTTGGATGGGAATGCATAACCTCACTAC	ACGTTGGATGTCTACCCCCAGACGAAAACT
rs3130048	ACGTTGGATGCAAAAAAACACACATTGCAAC	ACGTTGGATGTATCACCTTCTCTGTAAGGG
rs9276982	ACGTTGGATGATTTGGAACCAGAGACCCGC	ACGTTGGATGTAGGCCATGTGTCAAAGACC
rs4713466	ACGTTGGATGCTAATACATCATGCCTTGAG	ACGTTGGATGGGGCATTTGATCAAAGGGAC
rs986522	ACGTTGGATGTCCTCCAGTTTCCATTCTGC	ACGTTGGATGATCTCGGGCATGTTTGTTCC
rs2009658	ACGTTGGATGGATAATACCAACTTGTCACC	ACGTTGGATGTCCAACCCCTCTAACACTCT

PF: forward primer; PR; reveres primer.

**Table 6:** The primers of the 17 SNPs used for the extension step of MassARRAY.

SNP_ID	UEP_SEQ	EXT1_SEQ	EXT2_SEQ	EXT3_SEQ
rs5009448	CCTTAGGTGGCCTGT	CCTTAGGTGGCCTGTA	CCTTAGGTGGCCTGTG	
rs2523958	CCTCACGGTGCTGTCC	CCTCACGGTGCTGTCCC	CCTCACGGTGCTGTCCT	
rs2518028	CGTAGGAAGTGGGAAC	CGTAGGAAGTGGGAACC	CGTAGGAAGTGGGAACT	
rs435766	GGGTCAGCAGAGCTCGG	GGGTCAGCAGAGCTCGGC	GGGTCAGCAGAGCTCGGT	
rs213210	GCTTTAGCCTTCTAGTCC	GCTTTAGCCTTCTAGTCCC	GCTTTAGCCTTCTAGTCCT	
rs107822	cTTTCCGCGTGGTGTCTC	cTTTCCGCGTGGTGTCTCC	cTTTCCGCGTGGTGTCTCT	
rs17220087	gggCTCTGGAACGGCTGT	gggCTCTGGAACGGCTGTA	gggCTCTGGAACGGCTGTG	gggCTCTGGAACGGCTGTT
rs1536215	CTTGGTTTTCTGGTATGTC	CTTGGTTTTCTGGTATGTCC	CTTGGTTTTCTGGTATGTCG	
rs2070120	ctCCAGAACATTGACCTCAT	ctCCAGAACATTGACCTCATA	ctCCAGAACATTGACCTCATG	
rs79327197	ggcgGGTAAAACCTGCTCCA	ggcgGGTAAAACCTGCTCCAA	ggcgGGTAAAACCTGCTCCAG	
rs111394117	gggaTCTCCTAGGGGTCTTG	gggaTCTCCTAGGGGTCTTGA	gggaTCTCCTAGGGGTCTTGG	
rs3130048	cACACACATTGCAACAAAACA	cACACACATTGCAACAAAACAA	cACACACATTGCAACAAAACAG	
rs9276982	cCCCGCATGATTTCCTAGCTCC	cCCCGCATGATTTCCTAGCTCCC	cCCCGCATGATTTCCTAGCTCCT	
rs4713466	TCCCAAGTCAAAGGATTTTTAT	TCCCAAGTCAAAGGATTTTTATC	TCCCAAGTCAAAGGATTTTTATT	
rs986522	gtcaATTCTGCTTTGTCAGTAAC	gtcaATTCTGCTTTGTCAGTAACC	gtcaATTCTGCTTTGTCAGTAACG	
rs2009658	cCCTCAAATATTATTACTGCTACT	cCCTCAAATATTATTACTGCTACTC	cCCTCAAATATTATTACTGCTACTG	
rs139791445	GACTTGGTGATTTTTTTTTTTTCT	GACTTGGTGATTTTTTTTTTTTCTC	GACTTGGTGATTTTTTTTTTTTCTG	

UEP_SEQ: unextended primer sequence; EXT1_SEQ: extension primer sequence 1; EXT2_SEQ: extension primer sequence 2; EXT3_SEQ: extension primer sequence 3.

## Supplementary Information

Supplementary Information.

## Data Availability

The datasets analyzed during the current study are available in the dbSNP repository, (http://www.ncbi.nlm.nih.gov/feed/rss.cgi?ChanKey=dbsnpnews).
